# Debriefing strategies for interprofessional simulation—a qualitative study

**DOI:** 10.1186/s41077-022-00214-3

**Published:** 2022-06-18

**Authors:** Catherine Holmes, Edward Mellanby

**Affiliations:** 1 Leeds Teaching Hospitals Trust, Leeds, UK; 2 NHS Lothian, Edinburgh, UK

**Keywords:** Simulation, Interprofessional, Debriefing

## Abstract

**Background:**

Interprofessional education is becoming more common worldwide. Simulation is one format in which this can effectively take place. The debriefing after the simulation is a critical part of the simulation process as it allows reflection and discussion of concepts that arose during the simulation. Debriefing has been noted to be challenging in the literature. Debriefing after interprofessional simulation (IPS) is likely to have even more challenges, many related to the different backgrounds (profession, specialty) of the learners. This study was designed to investigate: ‘How do differing learner professions impact on delivery of post simulation debriefing after team based interprofessional simulation—what are the challenges and what strategies can be used to overcome them?’

**Methods:**

An initial review of the literature was used to identify current understanding and potential themes requiring further exploration. Using the results from the literature as a starting point for topics and questions to be asked, semi-structured interviews were planned, with those who are experienced in debriefing after IPS. The interviews were transcribed then analysed using a framework analysis.

**Results:**

The literature search resulted in twenty relevant papers. Four dimensions were drawn out from these papers that were directly related to debriefing after IPS: ‘the debriefer’, ‘method of debriefing’, ‘the learner’ and ‘psychological safety’. Sixteen interviews occurred between June and August 2020. Ten themes were extracted from the analysis of the transcripts of these interviews: number and specialty of debriefers, credibility, assumptions/preconceptions, nurses vs doctors, method of debriefing, the learner, hierarchy, safe learning environment, inclusion of all learners, and number of debriefers. These themes were fitted in the four dimensions identified in the literature search, and discussed as so.

**Conclusion:**

Several challenges and strategies were identified during this study. ‘It depends’ was a common answer received in the interviews suggesting that there is very little advice that can be given that applies to every situation. The main recommendation from this study is the support for an interprofessional group of debriefers in IPS although this does introduce its own challenges. Further research is suggested around the hierarchy found in IPS debriefing and how this translates to and from clinical practice.

**Supplementary Information:**

The online version contains supplementary material available at 10.1186/s41077-022-00214-3.

## Background

Interprofessional education (IPE) is increasing in popularity worldwide [1]. The World Health Organisation (WHO) suggests that ‘interprofessional education occurs when students from two or more professions learn about, from and with each other to enable effective collaboration and improve health outcomes. ’[2]. Publications and meta-analyses from organisations such as WHO, Cochrane and Best Evidence Medical Education [1–3] have explored this topic at length. These demonstrate that IPE can improve patient outcomes and therefore should be used across the world in healthcare settings but suggest that there are many research gaps that require filling to prove that IPE is more beneficial than single-profession education. Simulation is one of many methods within which IPE can occur [1] and has become increasingly commonplace in medical education [4, 5].

Following a simulated scenario, the debriefing is recognised as a critical but challenging aspect of the simulation process. There is a wealth of literature investigating the challenges and strategy of debriefing in general, some of which could be applied to debriefing after interprofessional simulation (IPS) but are not specifically designed for this. Examples are: ‘debriefing with good judgement’, a specific debriefing technique [6]; co-debriefing, i.e. having more than one debriefer [7]; and ‘learner centred’ debriefing [8].

Debriefing after IPS is becoming more common because of the increase in simulation and IPE but there seems to be few studies looking into IPS debriefing specifically [9]. Debriefing after simulation in general is well-known to be challenging at times; this often relates to specific characteristics of individual learners [10]. In debriefing after IPS, having an interprofessional cohort of learners, all with different learning needs, suggest that these challenges in debriefing may be multiplied, particularly when balancing the interprofessional learning objectives with the individual learner needs.

This study has therefore been designed to answer:

‘How do differing learner professions impact on delivery of post simulation debriefing after team based interprofessional simulation – what are the challenges and what strategies can be used to overcome them?’

Specifically, the aims are to

- Explore what the additional challenges are when debriefing an interprofessional group of learners after a simulation.
- Investigate any strategies recommended to overcome these challenges when debriefing an interprofessional group of learners.

## Methods

The design of this study had two approaches, a review of the literature followed by semi-structured interviews. The results from the literature were used to guide the interview discussion topics and influence the subsequent analysis of the data.

### Review of the literature

The aim of this literature search was to examine the existing literature on debriefing after IPS, identify gaps in research and, as per the question and aims for this study above, explore any challenges or strategies to debriefing after IPS. This was not a formal systematic or scoping literature review, but an informal process to inform the next stage of the study.

**Fig. 1:**
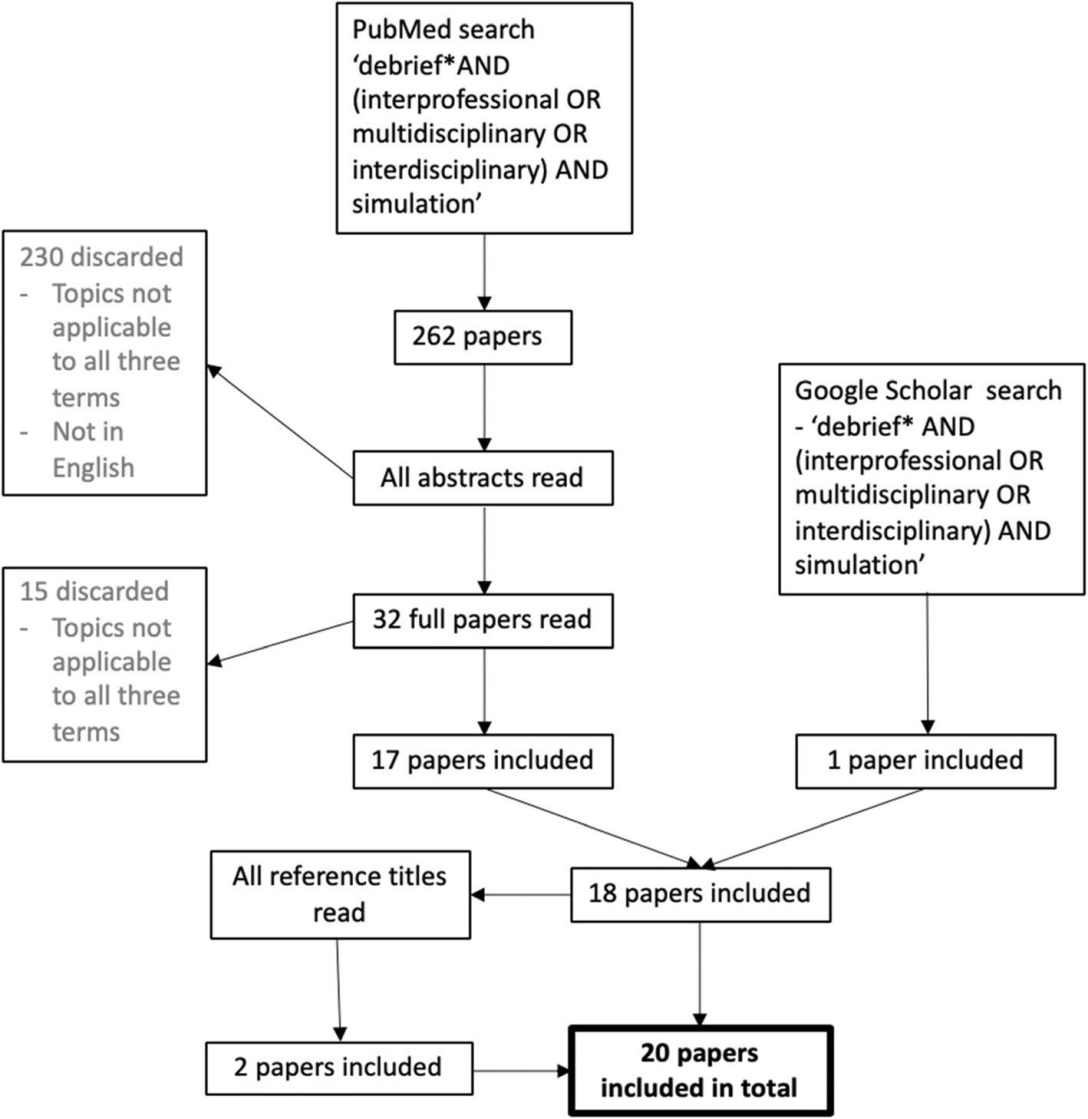
Literature search

The main concept explored in this research study was debriefing after simulation specifically in IPE. The search terms used for the literature search in November 2019 were therefore: ‘debrief* AND (interprofessional OR multidisciplinary OR interdisciplinary) AND simulation’. The ‘PubMed’ database was used along with a further search on the ‘Google Scholar’ search engine. Inclusion criteria included any paper that referred to debriefing after an IPS, even briefly. Any papers that did not include this were excluded. See Fig. 1 for the flowchart of the literature search process. The included papers were read and analysed by one of the authors (CH).

### Interviews

#### Research paradigm

The question posed in this study is clearly looking for opinion and personal experience rather than fact, with the hopes that sharing and collating such opinions will aid others to continue constructing their own. There is no right and wrong. Methodology for this study has therefore been considered from a constructivist viewpoint [11].

#### Qualitative approach and framework analysis

Ng et al. [12] suggest that experiential phenomena (as in this study) would be best researched qualitatively. Thematic analysis is one of the most commonly used approaches to managing data in a qualitative study [12]. From a constructivist viewpoint, the interviews, data analysis and writing up of the results are all part of the process of qualitative research as they promote further reflection and analysis [12].

The authors are both active and regular facilitators and debriefers of both interprofessional and single profession simulations but relatively novice researchers. Both authors have undertaken training that included qualitative methodology and have some experience of this process.

All of the ‘Standards for Reporting Qualitative Research’ have been met [13].

A framework method of thematic analysis was used in this study as described by Gale et al. [14]. This 7-step approach was followed:

1. ‘Transcription’—this was done manually by one of the authors (CH).
2. ‘Familiarisation with the interview’—as the same author performed the interviews then transcribed them, familiarisation was achieved.
3. ‘Coding’—interesting themes were coded manually on the transcript using a colour and a descriptive term. This was done by CH with input and discussion throughout with the second author (EM).
4. ‘Developing a working analytical framework’—after the first 5 transcripts were coded inductively, it became clear that there were approximately 10 themes.
5. ‘Applying the analytical framework’—the rest of the transcripts were then deductively coded using these themes.
6. ‘Charting the data into the framework matrix’—a spreadsheet was created where the summary of each of the themes from each transcript was manually inputted.
7. ‘Interpreting the data’—the data under each theme was interpreted and analysed. It was noted that each of the topics fit under or was one of the four main themes noted in the literature. Both authors reviewed and discussed this interpretation.

#### Choice of method

Semi-structured interviews were chosen so that specific questions could be asked, leaving space for the interviewee to interpret them and answer based on their own experience [15]. The literature [15–17] supports interviews as an appropriate choice of method for a qualitative study. The interview should be a conversation between two people with similar interests and experiences to draw out information to answer the aims of the study. This ‘conversation’ is appropriate from a constructivist view.

#### Recruitment and interview process

##### Sampling of participants

Purposeful sampling was used to include debriefers from a variety of different professions and experiencing IPS in different contexts and locations. This was done through direct working, meeting at simulation conferences or simulation courses. This ensured a cohort of information rich participants [18]. There is some bias in this form of sampling if you approach research from a positivist standpoint. However, from a constructivist point of view, this is not a limitation for this study as the aim is not to prove any specific facts, only to get opinions and experience and identify potential areas for future research. All participants have had regular experience with debriefing after IPS although their setting may vary, e.g. simulation lab, in situ simulation and life support courses. Face-to-face request was followed up with an email formally inviting them to take part with further information attached.

Interviewing to saturation is a concept that was considered—this entails stopping interview once all themes become recurrent and there are no new themes [12]. This was monitored when the data was analysed after each interview. Saturation of themes occurred around interview ten. However, the interviews continued to sixteen to try and ensure a more interprofessional representation of participants.

##### Consent

A written information sheet was provided to the participants (Appendix 3) Written consent was gained from each participant prior to initiating the interview (Appendix 4). The signed consent forms were uploaded to a secure online data storage area and any paper copies destroyed.

##### Interview schedule

The interview schedule was prepared beforehand. The first interview had questions written based on the experiences of the author, the information found in the literature search and the gaps identified in the literature search, all with the aims of answering the research question. The research question was read out to the participants first with an initial open question before further potential questions to narrow down focus depending on the topics brought up by the participants themselves. The interviews were semi-structured, i.e. there was scope to explore new ideas suggested by participants rather than going through every suggested question. The questions used in the interviews as a guide are in Appendix 5—while it was an iterative process with some changes in focus between interviews, new topics and questions were not formally written to avoid focussing too much on specific questions and less on the opinion of the participant.

A trial face-to-face interview took place in January 2020 which was included in the analysis with the consent of the participant. The rest of the interviews took place between June and August 2020.

##### Location

The interviews were initially planned to take place face-to-face; however, when the COVID-19 pandemic occurred, most were done via Microsoft Teams to comply with social distancing measures—this was not considered to affect the aims or objectives of the study.

##### Data collection

They were audio-recorded via dictaphone and audio data was stored securely in an online data storage area with an identifying number rather than a name.

The collected interview data was transcribed in a non-verbatim way (i.e. not writing down items like ‘erm’, ‘ok’, ‘yeah’ when they are not used in a meaningful way). This study was aiming to pick up themes of discussion and opinion rather than closely analyse the speech and conversation style so verbatim transcription was deemed unnecessary. A constructivist viewpoint aligns with transcribing and analysing the data—this will allow reflection and continuous development of the information received from the interviews with the aim to create a summary of this information in a format that can be shared.

Once transcribed, the audio files were deleted. The transcription was then anonymous. This transcription was only visible to the research team (or the participant if requested by them).

##### Project approval

Approval from the University of Edinburgh Medical Education Ethics committee was sought and received in January 2020 (see Appendix 1).

Approval from Health Research Authority (HRA) was sought and was received in March 2020 (see Appendix 2). Each NHS trust which has potential participants working for them received the appropriate documents to approve and sign as per the HRA process prior to initiating formal contact with the participants.

## Results

### Review of the literature

Twenty papers were included, all from 2013 to 2019. Each paper was read and the main topics which were discussed relating specifically to interprofessional debriefing, as per the aims of this study, were noted. This data is included in Appendix 6 along with a summary of the aim, design and methodology of each of the papers. These topics were collated under four main theme headings the debriefer, the method of debriefing, the learner and psychological safety.

Appendix 7 describes the demographics of the papers included. Appendix 8 shows whether the individual papers included discusses each of these four main themes.

See Table 1 for a summary of the results from the literature search. Perceived gaps in the literature have also been noted in Table 1.

**Table 1:** Summary of challenges, strategies and gaps identified by the literature search

Theme	Challenges:	Strategies:	Gaps:
The Debriefer [19–27]	• Larger group of debriefers	• Lead debriefer • Having more than one debriefer	• What are the challenges of having more than one debriefer? • Is there benefit to having an interprofessional debriefing team? • Why does the number or profession of debriefers make a difference? • What other strategies can help with this larger group of debriefers?
Method of Debriefing [9, 21, 27–32]	• Multiple debriefing tools • Interprofessional learners	• Having learner centred group discussion as main style of debriefing rather than direct feedback from debriefer(s)	• Should we be advising a specific debriefing tool for IPS? • Is it more challenging to stimulate group discussion with an interprofessional group of learners?
The Learners [21, 28, 33–35]	• Potentially having larger groups of learners in IPS	• Having a debriefing structure	• Are there larger groups of learners in IPS usually? • How do we ensure ‘interprofessional learning outcomes’ are met as well as the individual learner’s needs?
Psychological Safety [21, 25, 28, 30, 31, 36]	• Psychological safety of learners • Hierarchy	• Possibly having multiple debriefers	• Does having more debriefers increase the psychological safety of learners? • Are debriefers aware of the problems relating to hierarchy in IPS?

#### Interviews

Twenty-two participants were invited and sixteen agreed and were included. Reasons for not participating were all clinical or annual leave commitments. These were from three National Health Service (NHS) trusts in England. See Table 2 for the breakdown of the background of the participants of this study.

**Table 2:** Background of study participants

Trust (numbers of participants from each trust)		Debriefing background		Specialty and profession		
Trust A	12	In situ	15	Emergency department	11	1 advanced clinical practitioner 9 consultants 1 registrar
Trust B	3	Simulation lab-based (one profession/grade)	11	Medical education	3	1 retired consultant 2 nurses
Trust C	1	Life support courses	8	Anaesthetics	1	Consultant
		Simulation lab-based (interprofessional)	6	Neonatology	1	Consultant
		Other, e.g. non-clinical simulation, pre-hospital simulation, exam simulation	4			

The 16 interviews were completed between June and August 2020. The interviews lasted 30–70 min. Following the analysis process, four themes with nine subthemes were identified. It should be noted that ‘number of debriefers’ was relevant to both ‘the debriefer’ and ‘psychological safety’ so has been included twice.

These themes and subheadings are: ‘the debriefer’ (number and specialty of debriefers, credibility, assumptions/preconceptions, nurses vs doctors,), ‘method of debriefing’, ‘the learner’ and ‘psychological safety’ (hierarchy, safe learning environment, inclusion of all learners, number of debriefers).

It is important to point out that for the great majority of the opinions shared, a caveat of ‘*it* depends’ (e.g. on the learning outcome, or on the learners’ backgrounds, or on the debriefers’ style etc.) or similar was uttered by each of the participants so while a discussion around these collective opinions will now take place, there is no ‘correct answer’ advised for any particular situation. One participant particularly focussed on this, giving ‘it depends’ as an initial answer to every question asked before qualifying further.

Interestingly, many of the participants were very reflective upon their own debriefing practices throughout the interview, using examples and stories from their previous experiences to demonstrate their meaning when discussing debriefing practices. Several of them took aspects that were discussed in the interviews back to their educational practice to further develop after this reflection and discussion, demonstrating the importance of reflection and continuous professional development for even experienced educators and debriefers.

A discussion of the main themes and subheadings and how they relate to the literature will now follow.

### The debriefer

#### Number and specialty of debriefers

The interviews overwhelmingly suggested that having more than one debriefer from differing professions was perceived to have benefits in debriefing after IPS—all participants stated this. Having multiple debriefers in debriefing after simulation is not a new concept. Cheng et al. [7] highlight the benefits and challenges of co-debriefing. In their conclusion, they write ‘whether and how co-debriefing strategies need to be adapted in an interprofessional context is an important area of study’.

Collectively, the interview participants in this current study suggested that between 2 and 4 debriefers was optimum and having a debriefer with a nursing background debriefing alongside one with a medical background is beneficial for those IPS involving both these professions. This mimics the interprofessional working expected from the participants in the simulations (and clinical practice) and encourages learners of each specialty to engage. This is supported by the literature with Stockert et al. [24] stating that an interprofessional debriefing team can ‘model interprofessional behaviours and collaborative practice for the learners’.

However, this can become excessive in large multispecialty simulation debriefing sessions with more than four professions/specialties, e.g. trauma team. *‘Too many cooks’* was suggested as a direct quote by more than one participant. The participants also suggested that debriefing with others can be challenging, potentially those that you do not know well or who have limited training or experience in debriefing. It can lead to a difficult debriefing sessions with poor flow and interruptions from other debriefers because ‘everybody debriefs slightly differently, everybody has a slightly different focus, different speeds and different process for how you do it’. Strategies suggested to get around this included:

- Having a lead or ‘chair’ debriefer that brings in others at appropriate times or for specific sections—this ensures that the debriefing flows well and there is no competition amongst the debriefers to get their opinion in. This is backed up by Hull et al. [23]
- Planning how each debriefer will be involved in the debriefing helps to ensure that all areas are covered in an effective way and to mitigate debriefers having ‘completely different styles’.

#### Credibility

Credibility of the debriefer, or the perception of it, was a theme that featured in multiple interviews. This was summarised nicely by one of the participants: ‘The biggest sort of headline challenge would be credibility and natural ability to link to the different professional people within the team’. Interestingly, this was not particularly noted in the literature found in the brief literature search as part of this study, suggesting further investigation is needed for this topic in IPS. Clinical credibility is a complex topic, meaning different things to different people. A systematic review into clinical credibility in nursing has confirmed this—they were unable to find one clear definition [37]. In this study, while a specific level of seniority was not suggested by most with one participant stating ‘it's not about level it's almost about what background experience you have’, one participant felt that it was important that for clinical topics, the debriefer was more senior than the learners or at least supervised by someone more senior to ensure an element of clinical credibility. Some of the participants were lacking in clinically credible people with appropriate debriefing experience or training which they found challenging. This ‘debriefer credibility’ was perceived to be as important as clinical credibility as demonstrated by ‘I don’t think it needs to be the most senior person in the room, it needs somebody with debriefing skills.’ However, many experienced debriefers have never attended a debriefing course (including 6 out of 15 in this study) and it is difficult to define what is needed for debriefer credibility—something that was not clearly answered by this study. When discussing debriefer training or credibility, two things were mentioned by multiple participants, including all six participants who had not had formal debriefing training—a debriefing course as a starting point and, probably more importantly, watching and practising debriefing. Nearly all of the participants had debriefing coaching or ‘debriefing the debriefer’ built into their simulation programmes. Several participants suggested that debriefing training, particularly for those with the required clinical credibility, was key to improving debriefing after IPS. One participant mentioned that ‘building a community of practice’ or a ‘gang of debriefers’ in the trust should be the aim. One participant suggested as a solution to having both clinical and debriefing credibility present: ‘I think having a lead debriefer with a support structure of debriefers from different professions can add credibility’. This links back to the lead debriefer and interprofessional debriefing team discussion in the section above.

#### Assumptions/preconceptions

A further debriefer oriented point that came up repeatedly was around assumptions or preconceptions of the behaviours of the learners. There were several discussions around the assumed behaviour of learners based on their specialty (particularly if different to the debriefer), with the assumptions related to how the individual learner would engage in the debriefing based on their profession alone. Suggestions were that surgeons were less likely to engage or receive feedback well. Most participants recognised these preconceptions as such but there was a divide, some believing that they usually are accurate based on their experiences while others acknowledging that learners should be treated as individuals and keeping an open mind as the debriefer is key. This all suggests that many (if not all) have preconceived ideas and judge people based on many factors including their specialty, something that should be actively avoided once awareness of this is raised.

#### Nurses vs doctors

Comparison of doctors and nurses, their background training and experience of simulation as well as their behaviours in the debriefing, was discussed. Nearly all the participants commented on the differences between these two professions, many in their first response to an open question, suggesting that it is a key issue. Several of the participants who are doctors stated that they felt like they did not have the appropriate background, experience or understanding of what background knowledge the nursing staff should have or what level they should be working at, i.e. were not credible to be debriefing nurses—one participant stated that it is ‘arrogant’ of doctors to believe that they have that credibility and another acknowledging it is easy to ‘be a bit medic-centric’ (i.e. more focussed on medical rather than nursing staff) when debriefing. All of the nurses interviewed found it challenging to debriefing groups including doctors initially, with feelings that they did not have the credibility to do so. As their debriefing experience has increased, this was found to be less of a problem with the realisation that their knowledge and experience of non-technical skills (NTS) and debriefing outweighs that of most doctors.

On the other hand, some participants suggested that it was more a matter of trust and respect of whoever is debriefing regardless of their profession and that debriefing skill and experience outweighs the clinical background. It was suggested that doctors and nurses have different experiences of simulation in their education—‘the nurses tend to have done less simulation so are more nervous and anxious of the debrief and so you kind of have to be a little bit gentler’. Interestingly, it was thought also by a debriefer with a nursing background that the doctors are ‘too nice’ to the junior nurses and ‘tiptoe around them’ which leads to a poorer learning experience for them. These perceptions seem to stem from the assumptions of background knowledge and feelings and need to be addressed. Several suggested (from both doctor and nursing background) that having a nurse debriefing with the doctor(s) (i.e. an interprofessional debriefing team) would help with these perceptions and ensure appropriate level of learning is taking place. However, there were difficulties in finding available and trained debriefers amongst the nursing staff. Barriers to nursing staff participating in simulation included difficulties getting nurses protected education time to involve them as learners but also as debriefers. Doctors tended to have protected time and were more able participate. It was suggested by one participant that there was no recognition from ‘the system’ that interprofessional education is important and requires time, money and people.

It is important to mention that while the difficulties around nursing involvement have been discussed, there are other professions that also have similar barriers—five papers [22, 24, 33, 34, 36] from the brief literature search which mentioned or discussed debriefing after IPS include participants and/or debriefers from other professions, e.g. physical therapy and pharmacy backgrounds. None of these were from the UK and none of these other professions were mentioned in the interviews in this study at all (although it should be noted that the participants in this study either had a doctor or nursing background). This suggests that there is space to look into and move forward with other professions in IPS.

Table 3 summarises all of the challenges and strategies discussed around the topic of ‘the debriefer’.

**Table 3:** Challenges and strategies around ‘the debriefer’

Challenges	Strategies
Number of debriefers • Increasing number of debriefers • Debriefing with those without adequate debriefing skills • Debriefing with those that you do not know well	• Having a lead or ‘chair’ debriefer • Planning the debriefing amongst the debriefing team prior to the debriefing
Credibility of debriefer—clinical and debriefing	• Balancing clinical and debriefing credibility—an individual may not have both but you need someone with each in an interprofessional debriefing—having an interprofessional debriefing team may help with this. • Debriefing training • Ongoing coaching and mentoring of debriefers including ‘debrief of the debrief’
Automatic assumptions and preconceptions of debriefer towards the learners	• Ensuring awareness of this is raised in training and ongoing debriefer development • Encouraging reflection on the part of the debriefers • Having debriefer from each specialty can assist with getting around this.
Barriers to getting nursing staff involved in simulation and debriefing	• Limited immediate suggestion—requires work at a trust and national level including at universities during nursing training • This primarily highlights the lack of information of other ‘non-doctor’ professions.

### The learner

Having learners in IPS with different backgrounds was perceived to introduce challenges to debriefing an interprofessional group. These different backgrounds may be different professions, e.g. nursing or medical training, or different grades from students up to consultant or senior nurse level. ‘Everyone is at a different level’ was one answer when asked generically about the challenges around debriefing after IPS and many of the participants concurred with this. The differing learner backgrounds can cause complexities around what the learning outcomes for the session should be. For any IPS, most (if not all) of the learning outcomes are team based (often based around NTS according to the interview participants). Despite this, the learners still all have individual learning needs. Park and Holtschneider [35] write in their paper that aiming questions in debriefing towards team behaviours and actions rather than individual clinical behaviours ensures that interprofessional learning objectives are met. On a similar note, when asked specifically about tactics to get around this, the consensus from the interviews from this current study supported leaving very individualised questions and learning for after the debriefing, either a one-on-one conversation or via email. However, several suggested that most learning in these debriefing sessions should be applicable to all even if it was only related to the tasks of one profession. One participant advised: ‘you’ve just got to pause for a minute before you debrief and make sure that you are going to talk about stuff that’s relevant to everybody or an equal balance’. Another participant said: ‘because we are a complete team, we need to know about all the different cogs in it’. This is likely to be situation dependant however (along with most of the concepts discussed here)—ensuring that we are not devaluing or excluding learners by discussing things which are irrelevant to them is important. Interestingly there was no mention of interprofessional learning outcomes vs individual learner needs in the brief literature search (see Table 1)—potentially a topic that requires further exploration in IPS.

The interview participants acknowledged that most interprofessional debriefing sessions have a larger number of learners than those in single profession debriefings. This lead to increased challenges with every aspect of debriefing, in particular having more individual agendas to manage. It can lead to difficulties with the debriefer talking too much or becoming more ‘feedback’ focussed rather than debriefing—i.e. going away from learner-centred learning which has been advocated for in debriefing in general by Cheng et al. [8]. One participant phrased this as: ‘I would try and pull out what their learning is as opposed to try and inflict my teaching’.

### Method of debriefing

Based on the previous paragraph, the debriefing session should be adjusted depending on several factors—learning outcomes, individual learner background and debriefer experience amongst other things. This highlights the difficulties in providing a clear structure or debriefing framework that is applicable for all interprofessional debriefing and suggests that this is not what we should be looking for. ‘Debriefing with good judgement’ [6] is a strategy (rather than a framework) mentioned in the literature around debriefing after IPS [20–22, 26, 31, 33], suggesting that this is a useful tool. However, most interview participants stated that they had their own way of doing things, often based on frameworks (those mentioned include the Resuscitation Council’s ‘learning conversation’ [38], the ‘Diamond’ model [39], and a local framework base on the ‘PEARLS’ framework [40]) with small amendments that they developed over time. One technique described was ‘pre-briefing’ before the simulation or debriefing—to explain why the simulation is happening, that it is not a test, that it is about the team and system rather than individuals. This can avoid the perception that the simulation and debriefing is about a specific individual or group, with others there ‘to help’ rather than to learn. ‘Self-debriefing’ was another technique described by several of the participants. This involved the learners having a 5–10-min chat amongst themselves with no debriefer to write down some of the learning points that they want to discuss in the debriefing session. This ensured the debriefing was learner-centred and allowed the learners to relax and become familiar talking in an unfamiliar group. Most of the participants felt that giving the learners time to relax and calm down was beneficial. However, one participant was keen on getting the learners into the main debriefing session as soon as possible and talking while they were still ‘hot’, using this to get them to open up. This suggests there are several different techniques that may work for different debriefers and learners and further reinforces the point that there is no right or wrong answer. There was also an agreement that keeping the number of learning points discussed in the simulation to an achievable number (examples from 3 to 6 given) avoids overloading the learners and gave them the chance to meaningfully explore some key points.

Table 4 summarises the challenges (mostly found under ‘the learner’) and strategies found around both the ‘method of debriefing’ and ‘the learner’.

**Table 4:** Challenges and strategies around ‘method of debriefing’ and ‘the learner’

Challenges	Strategies
Individual learner needs	No specific debrief framework recommended. Specific tactics in debrief structure (discussed in more detail below): • Brief or pre-brief given before debriefing to ensure that it was clear that all learners were welcomed to contribute and participate • ‘self-debrief’ at the start of the debriefing session • Avoid too many learning points in discussion
Team vs individual learning outcomes	
Ensuring all learners involved in debriefing	
Larger groups of learners	

### Psychological safety

#### Hierarchy

The introduction of potential hierarchy gradients in an interprofessional group has been highlighted by several participants as a key difference in debriefing after IPS compared to a single-profession group of learners.

Interestingly, very few participants had ever noticed any direct problems with it in one of their debriefing sessions but all acknowledged the potential for it. One participant more clearly acknowledged the existing hierarchy and stated: ‘I still think there is a greater importance put on to the medical professionals as opposed to the nursing profession’. Multiple participants mentioned the term ‘flat hierarchy’ while referring to their area of specialty, particularly in the ED and in theatres, and the possible reason they had not had any direct issues with hierarchy. This is a commonly used term which ‘acknowledges that the contributions and opinions of all team members are crucial’ [41]. Several of the participants suggested that hierarchy may be more of a problem in other specialties, particularly surgery—this links back to assumed perceptions of specialties which may or may not be true—it is difficult to comment on this further here as there were no representatives from surgical specialties during this study—this may be an interesting avenue to explore further.

As all the interview participants are experienced debriefers and senior clinicians in their fields, it seems possible that their perceptions of hierarchy may not be the same as their learners, many of whom are likely to be more junior than them. To further explore this, information would need to be sought from these junior learners. Van Schaik et al. [36] interviewed the learners in their debriefing sessions and found that hierarchy limited the discussion—this is only one study from the USA however which has a different set up to the UK, further exploration of this in the UK would be interesting.

#### Safe learning environment

Creating a ‘safe learning environment’ was discussed in every interview as the primary technique used to acknowledge and ensure the psychological safety of the learners. Many participants referred to a ‘spiel’ that they run through at the start of the debriefing to ensure that all learners feel safe and included. This was usually part of the ‘pre-brief’ as discussed in the ‘Method of debriefing’ section. This was not specific to IPS, but several participants suggested that it helps to overcome some of the potential hierarchy issues which may be more challenging in IPS. Frequent things that were mentioned as being covered in this ‘spiel’ include: ensuring that all learners are aware not to discuss things about the simulation or the debriefing session ‘outside these four walls’; ensuring that all learners were aware that it was a team simulation and debriefing, not aimed at any individual; encouraging all learners to participate, regardless of grade or profession and making it clear that it was a learning experience and it was not expected to go perfectly—honest feedback and discussion would occur with the aim of learning, not an attack on anyone. In their paper, van Schaik et al. [36] did suggest that having this ‘safe environment’ was not enough to get around issues of hierarchy but it is unclear what else may be required.

The only other strategy suggested by the interview participants was the physical set up of the debriefing session, ensuring it was in a quiet private space with enough space for all the participants to sit and making sure that they were not stood or sat in their profession groups during the debriefing to avoid a physical emphasis of this hierarchy. While there is little new in this suggested strategy from these interviews, it does emphasise the need for further research around hierarchy and safe learning environments in IPS and debriefing.

#### Inclusion of all learners

Another challenge noted was around including all learners. There was a perception, suggested by several of the interview participants, that the quieter participants were so because they were worried about speaking in front of their colleagues or in a group and that this anxiety was likely to be heightened in more junior members of staff, particularly when more senior staff were present. It was also acknowledged by a few that this can be a personality trait of the learner and does not necessarily mean that they do not feel included or are not learning.

Asking more junior members of the team to provide a summary at the start of the debriefing session was one way around this suggested by many of the participants. Asking someone more junior to do this ensures that they felt involved and that their contributions were valued and expected as well as ensuring that the focus of the simulation and the debriefing was not on one particular individual (e.g. the team leader) but on the team as a whole.

There were some opposing opinions about asking questions directly to individual learners to draw them into the discussion. Several participants suggested that that was a good method to ensure that everyone was involved but caution was advised from other participants. They had some concern that it could cause discomfort to some learners, and they may feel like they were being put on the spot. It seems that this was something that the debriefer must decide at the time whether or not it will cause more harm than good based on their perception of the learner from the simulation.

#### Number of debriefers

The number of debriefers was a key discussion point in debriefing IPS. It has been included here again, because there was a clear sub-theme relating to the effect of more than one debriefer on the psychological safety of the learners from both the literature search and the interviews. There were suggestions that an increased number of debriefers can have both a positive and negative effect on the psychological safety of the learners. Paige et al. [25] suggested that increasing the number of debriefers increased the psychological safety of participants because it reduced the time taken to ‘establish a learning environment’ which was noted to be associated with the psychological safety of the learners. In one of the interviews of this current study, having more than one debriefer was also suggested to be of benefit if there is a particularly distressed learner that requires one on one support. This allowed the debriefers to split up and continue the main debriefing session as well as supporting a struggling learner.

However, several of our interview participants had concerns or experiences that increasing the number of debriefers could cause difficulties for the learners. This was related to the number of debriefers potentially outnumbering or appearing to overpower the learners. Another concern was that an increased number of debriefers from different specialties may become too focussed on their own specific clinical areas which may cause confusion amongst the learners and poor flow in the debriefing—linking back to the discussion on interprofessional versus individual learning outcomes.

Strategies noted by the interview participants to ensure the positive aspects were gained from multiple debriefers without adding the negative effects included having a lead debriefer, planning the debriefing amongst all the debriefers and keeping the number of learning points down (covered in ‘The debriefer’ section above). It was also noted that most of the participants advocated for 2–4 debriefers as a maximum so while suggesting more than one debriefer has benefits, limiting the number of debriefers somewhat appears to be another strategy to ensure that the psychological safety of the participants and the flow of the debriefing is optimised.

Table 5 summarised the findings liked to the topic of psychological safety.

**Table 5:** Challenges and strategies around ‘psychological safety’

Challenges	Strategies
Hierarchy	• Ensure ‘safe learning environment’ as pre brief • Physical set up of debriefing session *This may not be enough, further research into hierarchy is needed*.
Multiple debriefers	• Having a lead debriefer and planning debriefing (see ‘The debriefer’ session)
Inclusion of learners	• Ensure ‘safe learning environment’ as pre brief • Use of language and debriefing techniques

### Limitations

The primary limitation of this study is that while attempts were made to recruit an interprofessional group of participants, there was only partial success at this, with many of the participants being senior doctors, mostly in emergency medicine. There have been findings from them and the other participants that it would be helpful to explore with a wider interprofessional group. As discussed in the ‘Nurses vs. doctors’ section, it is possible that one of the reasons it was difficult to recruit nurses and other professions to this study centred around the potential barriers in them getting involved in simulation and debriefing.

Many of the participants, regardless of profession, have been mostly involved in interprofessional debriefing after in-situ simulation in particular. Several have also been involved in life support courses and other sim-lab-based sessions; however, many of the suggestions and conclusions are perhaps more applicable to the in-situ environment, given the participants’ experience, albeit some do appear to be transferrable.

An informal literature search was performed for background information. Both PubMed and Google Scholar were used to look for literature but other databases could have also been reviewed to ensure a more complete review of the literature. Other improvements could include having more than one author reviewing the full literature and performing a more formal scoping or systematic literature review.

A single author analysed and discussed the data found in the literature and interviews with review from and discussion with a second author. There may be views and opinions that may be individual specific. It is probable that with other viewpoints, it would be possible to create a richer discussion, having more than one person analysing the data was suggested by Gale et al. [14] whose framework was followed in this study. However, from a constructivist viewpoint, this study does provide options and opinions that are valid additions to the current literature.

## Conclusion

Multiple potential challenges as well as some possible strategies to overcome them have been identified from this study. Few of these answers are new information, most have been discussed in previous literature in other contexts. They have, however, been discussed here specifically in relation to IPS.

While there are several suggestions based on the research and opinions of the participants, the primary answer to many of the challenges and questions is, as stated multiple times by one of the interview participants, ‘it depends’. Many of the challenges and suggestions noted in this study are individual-dependent (either based on the learner or the debriefer) or situation-dependent, and it is important to remain flexible and acknowledge that there is no correct answer for every situation and there will never be. Personality and perceptions played a large part in many of the discussions suggesting that individuality and our behaviour as people is a key factor in all of this, something that cannot be easily ‘fixed’ or changed. Many of the behaviours and preconceptions involved in IPS mirror cultural issues from clinical practice which are commonly overlooked. Debriefing after IPS could provide a forum for discussion of these issues and potentially trigger change from the ‘top down’ and the ‘bottom up’ with a group of interprofessional individuals from a spectrum of grades.

One particular recommendation based on this study would be the use of an interprofessional debriefing faculty for IPS. This could help with having credibility from a clinical and debriefing aspect between the debriefers. It would also ‘role model’ interprofessional working that can hopefully be transferred back into clinical practice. There is a danger of faculty from the wrong specialty leading a debriefings session if they demonstrate they have not considered or understood the experience and backgrounds of the different professions—it could make them feel less valued and worsen hierarchy issues that likely already exist. There are also suggestions for adjustments to debriefing structure or framework to ensure that the interprofessional team is considered. Although there is not a specific debriefing framework to use, many individuals or departments have their own structure that they have developed, although these often require deviation from depending upon the learners and the situation. This confirms that a structure should be followed but there is not a ‘correct’ option.

Areas in which further research would be of particular interest include studies looking into hierarchy in debriefing after IPS from a debriefer and learner viewpoint. Investigating how to break these barriers down and how this hierarchy translates out of simulation to the workplace would be fascinating.

## Supplementary Information

**Additional file 1: Appendix 1**. Ethics committee approval.

**Additional file 2: Appendix 2**. HRA approval letter.

**Additional file 3: Appendix 3**. Participant information sheet.

**Additional file 4: Appendix 4**. Participant consent form*.*

**Additional file 5: Appendix 5**. Interview questions.

## Data Availability

The datasets generated and/or analysed during the current study are not publicly available due to requirement to delete audio data after transcription as it would not be anonymous. Written transcripts are available from the corresponding author on reasonable request.

## Appendix 6

Table 6

**Table 6:** Description of papers included in literature review

Main Author, Date	Aim(s)/Questions	Design	Terminology	Methods	Quantitative or qualitative	Summary of findings relating to debriefing
Boet, 2013 [20]	Is ‘within team’ debriefing equal to ‘instructor led’ debriefing?	Randomised controlled repeated measures design	Interprofessional	Teams randomised to ‘within team’ or instructor led debriefing. Videos of pre and post debrief scenarios assessed by 3 blinded assessors and a score created via a framework. Pre- and post-test scores compared.	Both (mostly quantitative data)	Both ‘within team’ debriefing and instructor led debriefing are effective at improving team performance.
Boet, 2014 [19]	12 tips with information on developing, implementing, assessing, and evaluating an interprofessional simulation-based education session.	Narrative	Interprofessional	Description—12 tips for interprofessional simulation. One of 12 tips is a paragraph on debriefing.	Qualitative	Interprofessional debriefs are challenging. Need to ensure all learners are involved as well as psychologically safe. There is no gold standard for method of debrief—some use single debriefers, others have debriefers from all specialties.
Boet, 2016 [21]	A narrative analysis on content of discussion during both ‘within team’ and ‘instructor led’ debriefing.	Exploratory case study	Interprofessional	Analysis of audio recordings of debriefs from debriefs—both ‘within team’ or ‘instructor led’. Data from a larger randomised controlled trial (Boet 2013 above).	Qualitative	There was no significant difference in the achievement of the learning outcomes in either group suggesting that ‘within team’ debriefing is a viable option.
Brown, 2018 [22]	To determine the best practices in interprofessional debriefing by comparing in person with tele-debriefing and single vs interprofessional debriefers	2 group quasi-experimental cohort comparative	Interprofessional	Questionnaires from all students after debriefing—average scores on 6 items on Likert scale found (> 4 is acceptable)	Quantitative	No significant difference between single and interprofessional debriefing (although single did have a higher score). Significantly higher score in in person debriefs compared with teledebrief.
Cheng, 2013 [26]	To determine whether use of a script designed to facilitate debriefings by novice instructors and/or simulator physical realism affects knowledge and team performance of learners in simulated cardiopulmonary arrests.	Experimental randomised controlled trial	Interprofessional	Randomised controlled trial—each team randomised to 1 of 4 groups (with or without scripted debrief and with high or low realism). Post scenario MCQs as well as Team Leader Behaviour Performance (BAT) and Team Clinical performance (CPT)	Quantitative	Scripted debriefs caused an improvement in knowledge (MCQ) and BAT scores but no significant difference in CPT scores compared to non-scripted debriefs.
Endacott, 2019 [27]	The main aims of this study were to identify: (1) frameworks used for debriefing interprofessional and uni-professional team-based simulations, (2) metrics that have been developed to assess the quality of debriefing and (3) evidence gaps for debrief decision	Systematic review	Interprofessional	Lit search PubMed, CINAHL, MEDLINE and Embase ‘Simulation’ AND (‘Debrief* OR Feedback’) AND ‘Evaluation’ AND (‘Quality OR Framework OR Method’)	Quantitative and qualitative	All used different debrief frameworks. Debrief framework improves debrief quality. ‘Some key aspects of debrief for team-based simulation, such as facilitator training, the inclusion of a reaction phase and the impact of learner characteristics on debrief outcomes, have no or limited evidence and provide opportunities for future research, particularly with interprofessional groups.’
Hull, 2017 [23]	‘We explored the value of 360° evaluation of interdisciplinary debriefing by examining learners’, debriefers’ and expert evaluators’ perceptions of the quality of debriefing.’	Exploratory, cross-sectional observational study	Interdisciplinary	Comparison of OSAD scores of learners, debriefers and expert debriefers of debriefs.	Quantitative	Debriefers themselves and learners tend to score the quality of debrief higher than the expert debriefers suggesting some overconfidence on the part of the debriefers which may mean that they don’t seek training opportunities. External evaluation of debriefs should be regularly completed to drive educational excellence.
Kolbe, 2013 [31]	To find out if TeamGAINS is an effective debrief tool	Survey (to validate debrief tool)	No term—‘team’ used instead	A ‘self-assessment’ of the debrief was completed by the learners. Specifically, psychological safety and ‘leader inclusiveness’ were scored by the learners.	Quantitative	Debriefing using the TeamGAINS method was effective based on learner assessment including increasing psychological safety and leadership inclusiveness among learners.
Meny, 2019 [33]	This study explored the difference in post-simulation reflections of multiple small groups compared to a single large group after an interprofessional simulation.	Cohort study	Interprofessional	Comparison of experiences for those in large group debrief (60 participants and those in small group debriefs (4-6)).	Quantitative and qualitative	There was no difference in the ability to identify an area for improvement or continued growth between those students participating in the large group debrief compared to the small group debrief section.
Nystrom, 2016 [28]	To explore debriefing as a practice intended to support students’ interprofessional learning	Observational	Interprofessional	Collaborative analysis of video-recorded debriefs		‘Two main methods of debriefing emerged ‘algorithm based’ and ‘laissez-faire’ neither ensured the main topic of interprofessional collaboration came up. Sociomaterial aspects such as time constraints, material set-up and social interaction affect as much as opportunity for reflection.’
Paige, 2019 [25]	Investigating whether differences exist for prebriefs (PBs) and debriefs (DBs) among faculty teams after a high-fidelity simulation-based training for interprofessional education of pre-licensure students and analysing potential causes for any differences in the quality of PBs and DBs.	Retrospective analysis of prospective collected videos of debriefs.	Interprofessional	Use of OSAD scoring to assess debrief videos after simulation—comparison of score changes over multiple debriefs/prebriefs.	Quantitative	‘In conclusion, effective debriefing is essential for learning in HF SBT. To date, the quality of such debriefing in actual teaching practice is not well known. The scores of all 7 teams were good. Faculty teams tended to improve the quality of their DBs through time.’
Park, 2016 [35]	Discussion of ‘debrief from the learner’s point of view’	Narrative column	Interprofessional	N/A	Qualitative	Using questions in the debriefing relating to team aspects and behaviour rather than individual clinical behaviour of specific team members can help focus the debrief on interprofessional interactions.
Poore, 2019 [9]	To present an interprofessional debriefing tool, the Debriefing Interprofessionally: Recognition and Reflection (DIPRR), designed to incorporate IPE into any simulation experience.	Survey (to validate debrief tool)	Interprofessional	Survey to obtain content validity index on each question in the debrief tool.	Quantitative	‘The ‘Debriefing Interprofessionally: Recognition and Reflection’ tool allows uni- and interprofessional simulation to be transformed into an IP learning opportunity.’
Richmond, 2017 [34]	This resource is an interactive, interprofessional, small-group activity designed for up to six participants per standardised patient.	Narrative	Interprofessional	Survey to participants—quantitative questions on a Likert scale and qualitative open answer questions.	Quantitative and qualitative	‘The experience has been successful at meeting interprofessional curricular goals. Students valued the interprofessional interaction and especially the debrief discussion about their experience.’
Van Schaik, 2015 [36]	To explore the residents’ perceptions of simulation-based interprofessional team training.	Qualitative analysis	Interprofessional	Analysis of interviews to examine paediatric residents’ self-assessment of team leadership skills during simulated resuscitations.	Qualitative	‘Interprofessional simulation-based team training offers an opportunity for residents to learn about, from and with other health care professionals but barriers exist that hamper its effectiveness—anxiety provoking as with colleagues that they work with normally, hesitant about providing honest feedback because of fear of offending.’
Schere, 2019 [30]	Description of implementing multidisciplinary team simulation from an interventional radiology point of view.	Narrative	Multidisciplinary	No method—just description of tool and why it is used.	Qualitative	‘In conclusion, multidisciplinary team simulation training is an extremely useful tool and future research will continue to demonstrate its importance in communication, technical skills, and therefore patient care.’
Stockert, 2017 [24]	‘To determine the prevalence of interprofessional simulation as an educational strategy for teaching IPE content, to identify the curricular objectives associated with interprofessional simulation experiences, and to characterize the instructional design features of interprofessional simulation experiences in entry-level PT education programs in the USA’	Cross-sectional descriptive study	Interprofessional	A survey was sent out to each centre that has a PT programme. Responses were analysed and provided mostly quantitative data.	Quantitative	‘In PT education programs that use immersive simulation for IPE, most programs conduct simulation experiences consistent with recognized best practice. In addition, nearly all programs that used immersive simulation for IPE included learning objectives related to the four IPEC competencies for promoting interprofessional collaborative practice.’
Sullivan, 2018 [29]	‘On which non-technical skills do teams perform the strongest (e.g., decision- making, communication, leadership, cooperation or stress management) during a trauma resuscitation simulation? Are improvements in non-technical skills observed immediately following a debriefing session? Does the debriefing appear to improve some skills more than others?’	Observational	Interprofessional	Scoring of team performance of non-technical skills via T NOTECHS score. Analysis of debrief strategies from video footage of debrief.	Quantitative and qualitative	‘Interprofessional team simulation in trauma resuscitation scenarios followed by debriefing differently impacted individual non-technical skills domains. The debriefings were primarily focused on directive performance feedback. Additional facilitation strategies may target other non-technical skills in different ways.
Thompson, 2018 [42]	Does the introduction of a written tool to help facilitate high-quality debriefing techniques improve the ratio of judgmental, non-judgmental, and good judgment statements from facilitators.	Quasi-experimental observational study	Interprofessional and interdisciplinary	Observation of videos of simulation debriefs before and after introduction of debrief tool	Quantitative	Debrief written tool increases number of ‘good judgement’ comments as part of debrief with a significant decrease in non-judgemental comments.
Yang, 2019 [32]	Introduction of describe, analysis, application (DAA) based integrated interprofessional collaboration and team efficiency (IIT) simulation model.	Narrative	Interprofessional and multiprofessional	No method—just description of tool and why it is used.		Taken together, in an attempt to ensure high-quality care delivery, the integrated IPC and team-efficiency intervention is a feasible and successful strategy for training multiprofessional trainees.

## Appendix 7

Table 7

**Table 7:** Demographics of papers from literature search

Author, date	Country	Clinical topic of simulation	Population	Undergraduate/postgraduate
Boet, 2013 [20]	Canada	Cardiac arrest in theatre	Surgical and anaesthetic trainees and operating room nurses.	Postgraduate
Boet, 2014 [19]	Canada	n/a	n/a	n/a
Boet, 2016 [21]	Canada	Cardiac arrest in theatre	Surgical and anaesthetic trainees and operating room nurses.	Postgraduate
Brown, 2018 [22]	USA	ACLS—students in sim lab	Senior critical care students in nursing, respiratory therapy and medicine.	Undergraduate
Cheng, 2013 [26]	USA	Paediatric life support	Novice instructors on paediatric life support courses, participants in paediatric life support course.	Postgraduate
Endacott, 2019 [27]	UK	n/a	n/a	n/a
Hull, 2017 [23]	UK	Medical emergencies	Debriefers of medical/nursing student interdisciplinary simulation	Undergraduate
Kolbe, 2013 [31]	Switzerland	CRM—critical events in anaesthesia	Anaesthetists and anaesthesia nurses	Postgraduate
Meny, 2019 [33]	USA	Pharmacy—discharge planning	Pharmacy students who have taken part in an interprofessional simulation	Undergraduate
Nystrom, 2016 [28]	Sweden	n/a	Medical and nursing students	Undergraduate
Paige, 2019 [25]	USA	Trauma	Medical and nursing students	Undergraduate
Park, 2016 [35]	USA	n/a	n/a	n/a
Poore, 2019 [9]	USA	n/a	Nursing education simulation experts with/without publications and some IP simulation experts from all healthcare professions.	n/a
Richmond, 2017 [34]	USA	Discharge planning	Interprofessional team of 4–6 students nursing, medicine, pharmacy, social work, and physical therapy	Undergraduate
Van Schaik, 2015 [36]	Germany	Paediatrics (cardiac arrest)	Paediatric residents and nursing staff (sometimes with pharmacists and medical students but not consistently)	Postgraduate
Schere, 2019 [30]	USA	n/a	n/a	n/a
Stockert, 2017 [24]	USA	n/a	Physical therapy programmes in the USA	Undergraduate
Sullivan, 2018 [29]	USA	Trauma	Teams of ED and surgical residents and ED nurses	Postgraduate
Thompson, 2018 [42]	USA	Trauma	Teams of five trauma trainees—multidisciplinary	Postgraduate
Yang, 2019 [32]	Taiwan	n/a	n/a	n/a

## Appendix 8

Table 8

**Table 8:** Topics covered in papers

Author, date	Primarily debrief focussed paper?	The Debriefers		The learners size of group of learners (see also ‘Population’ column in Appendix 2)	Debrief framework	Psychological aspect of debriefing
		Number of debriefers	Profession of debriefers			
Boet, 2013 [20]	Yes	Yes—0 vs 1	‘Formally trained’ debriefer. Profession not mentioned	3	‘Within team debriefing’ vs advocacy/inquiry	Not mentioned
Boet, 2014 [19]	No	Mentions very briefly (i.e. 1 debriefer, co-debriefers, within team debrief)	Mentions very briefly—i.e. could have one debriefer from each profession—as an option though not an opinion that this is right	Not mentioned	Not mentioned	Mentions very briefly
Boet, 2016 [21]	Yes	Yes—0 vs 1	‘formally trained’ debriefer—profession not mentioned	3	‘Within team debriefing’ vs Advocacy/inquiry	Not mentioned
Brown, 2018 [22]	Yes	1 vs 2	unknown	6-9 students	Advocacy/Inquiry	Very briefly mentioned
Cheng, 2013 [26]	Yes	1	‘Novice debriefers’ nurses, respiratory therapists, physicians	4–5	Debriefing script specifically written based on advocacy-inquiry theory	Not mentioned
Endacott, 2019 [27]	Yes	Not mentioned	Not mentioned	Not mentioned	Yes—multiple frameworks discusses	Mentions briefly
Hull, 2017 [23]	Yes	14/41 teaching sessions—1 debriefer 27/41 >/= 2 co-debriefers (always one lead debriefer)	When co-debriefed, i.e. more than one debriefed, had at least one physician and one nursing debriefer	4–8	Not mentioned	Not mentioned
Kolbe, 2013 [31]	Yes	2	Psychologist and anaesthesiologists	6	Team-GAINS—Guided team self-correction, advocacy-inquiry and systemic constructivist debriefing	Yes
Meny, 2019 [33]	No	4 in large groups, 1–2 in small groups	Each involved specialty in large group (pharmacy, medicine, nursing, physical therapy), 1–2 of differing specialties (2 when available)	‘Large’ 60 ‘Small’ 4–6	Advocacy/inquiry	Does not mention
Nystrom, 2016 [28]	Yes	1	‘varying health professionals’	4–6	Steinwachs debriefing	Yes
Paige, 2019 [25]	Yes	1–3	2 nurses and 2 doctors (one surgeon and one ‘internist’)	Does not mention	Not mentioned	Brief mention
Park, 2016 [35]	Yes	Not mentioned	Not mentioned	Does not mention	Not mentioned	Not mentioned
Poore, 2019 [9]	Yes	Not mentioned	Not mentioned	Does not mention	‘Debriefing Interprofessionally -Recognition and Reflection’ – main focus	Not mentioned
Richmond, 2017 [34]	No	Small group self- debrief first. ‘At least 1’ for large group debrief	Not mentioned	4-6 for small group self debrief. 15-20 in facilitated large group debrief	Not mentioned	Not mentioned
Van Schaik, 2015 [36]	No	2	One ‘MD’ and one RN	10-14	Not mentioned	Yes – discussion around anxieties around simulation and social identity affecting simulation
Schere, 2019 [30]	No	Not mentioned	Not mentioned—does suggest that all professions should be involved in organising simulation	Does not mention	Yes – suggests planning specific structured questions to ask during debrief	yes
Stockert, 2017 [24]	No	Yes – interprofessional debrief tea, (i.e. >1) in 51.1%, 40% had one or more debriefers from a single profession	Yes—advocates for interprofessional debriefing team	Does not mention	Not mentioned	Does not mention
Sullivan, 2018 [29]	No	3 (mixed faculty)	Trauma surgery, emergency medicine and emergency medicine nursing (all involved professions)	5	Yes—PEARLs	Does not mention
Thompson, 2018 [42]	Yes	3	Trauma attending, emergency Medicine attending, emergency medicine nurse	5	TEAM debrief tool – adapted from PEARLS	Does not mention
Yang, 2019 [32]	No	Not mentioned	Does not mention	Does not mention	Describe, analysis, application (DAA) based integrated interprofessional collaboration and team efficiency (IIT) model	Does not mention

## Abbreviations

HRA: Health Research Authority
IPE: Interprofessional Education
IPS: Interprofessional Simulation
NHS: National Health Service
NTS: Non-technical Skills
WHO: World Health Organisation
