# Supplementary figures and images for: Debriefing strategies for interprofessional simulation—a qualitative study
Source: Adv Simul (Lond). 2022 Jun 18;7:18. doi: 10.1186/s41077-022-00214-3 (PMC9206121; doi:10.1186/s41077-022-00214-3)

**
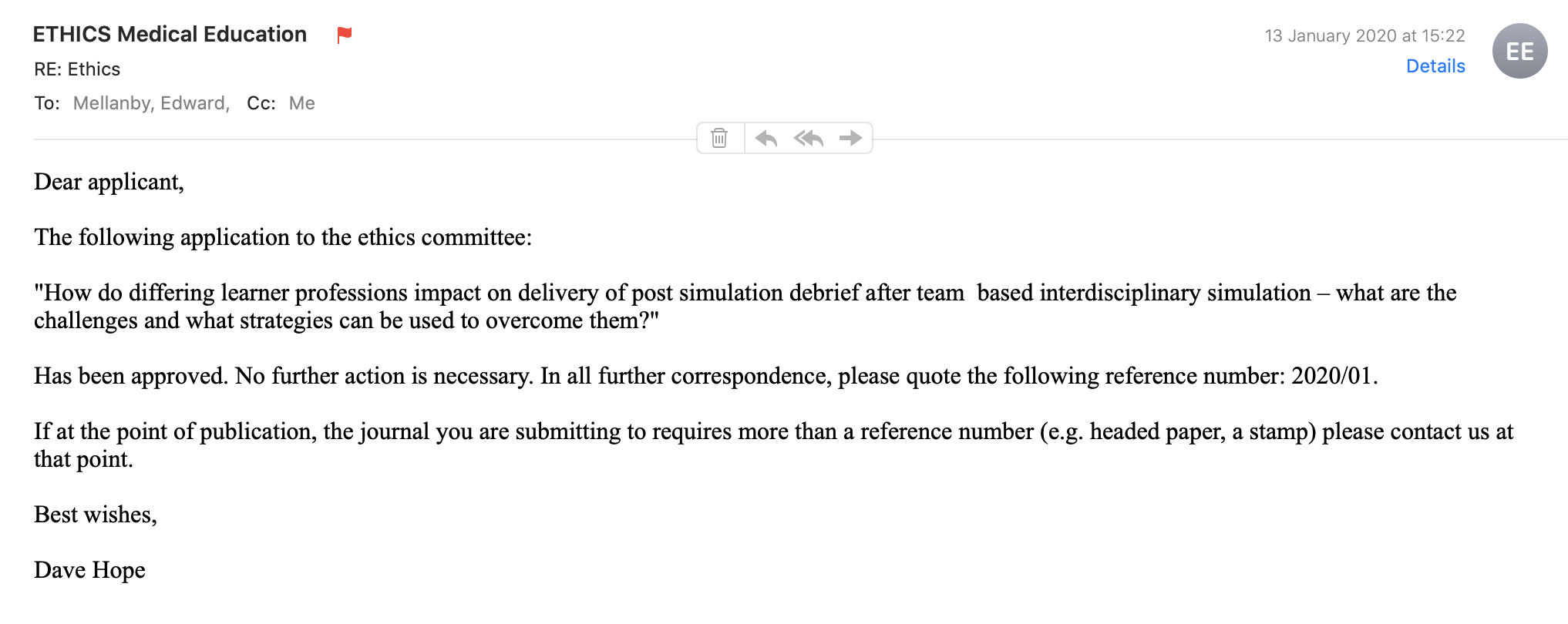
**

Supplement: Supplementary file 1 — Additional file 1: Appendix 1. Ethics committee approval. [file 41077_2022_214_MOESM1_ESM.docx]

**
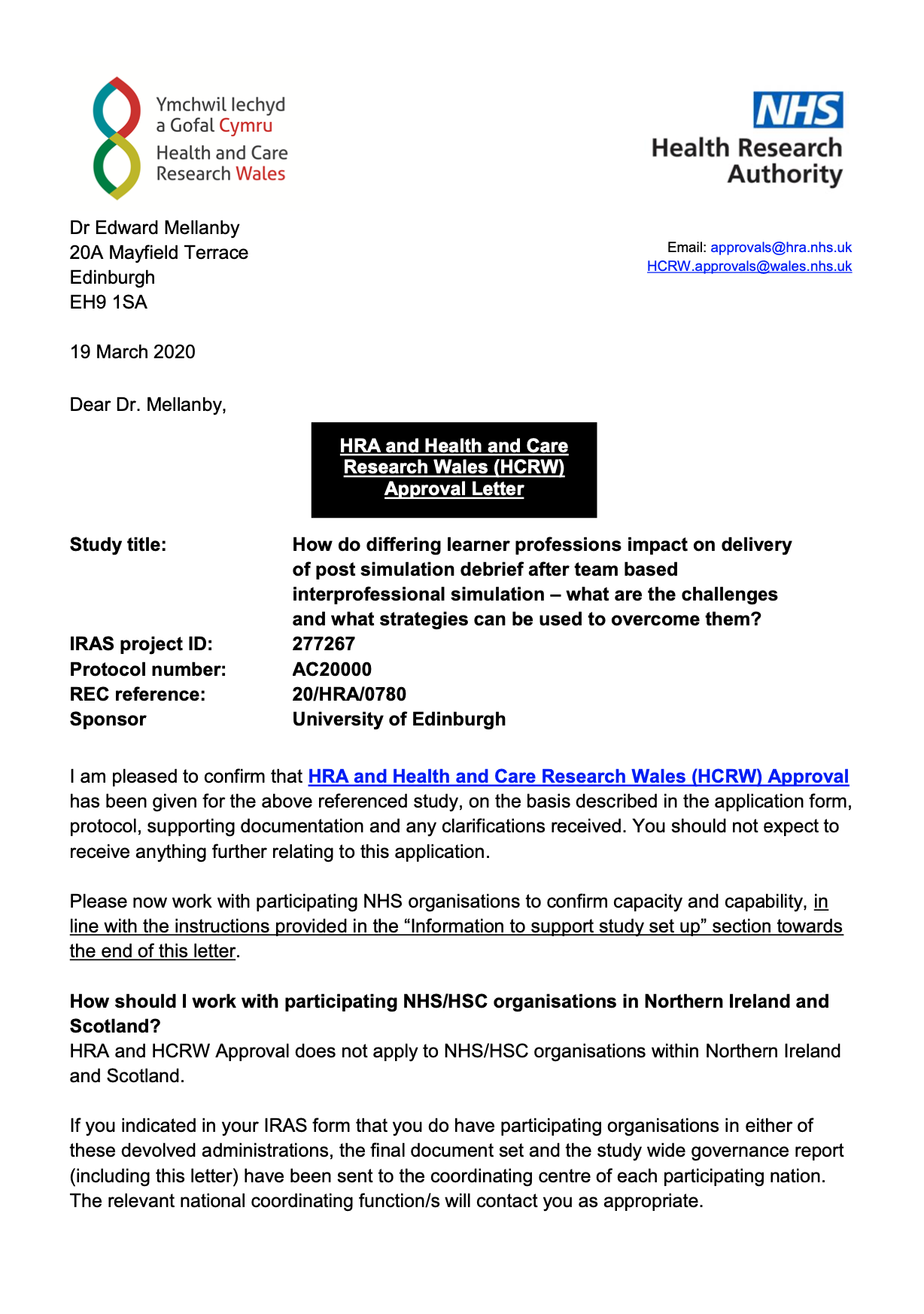
**

**
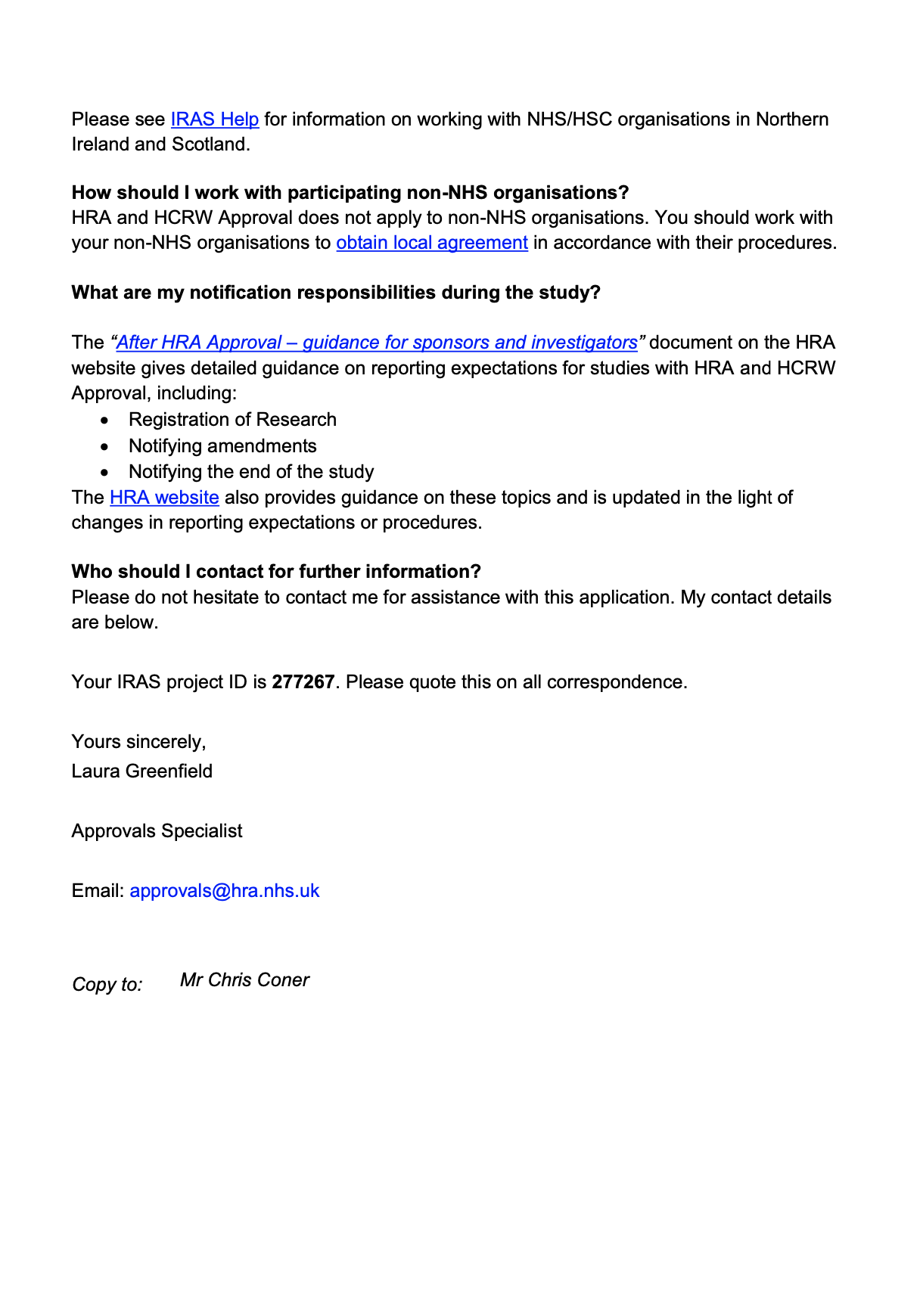
**

Supplement: Supplementary file 2 — Additional file 2: Appendix 2. HRA approval letter. [file 41077_2022_214_MOESM2_ESM.docx]

**
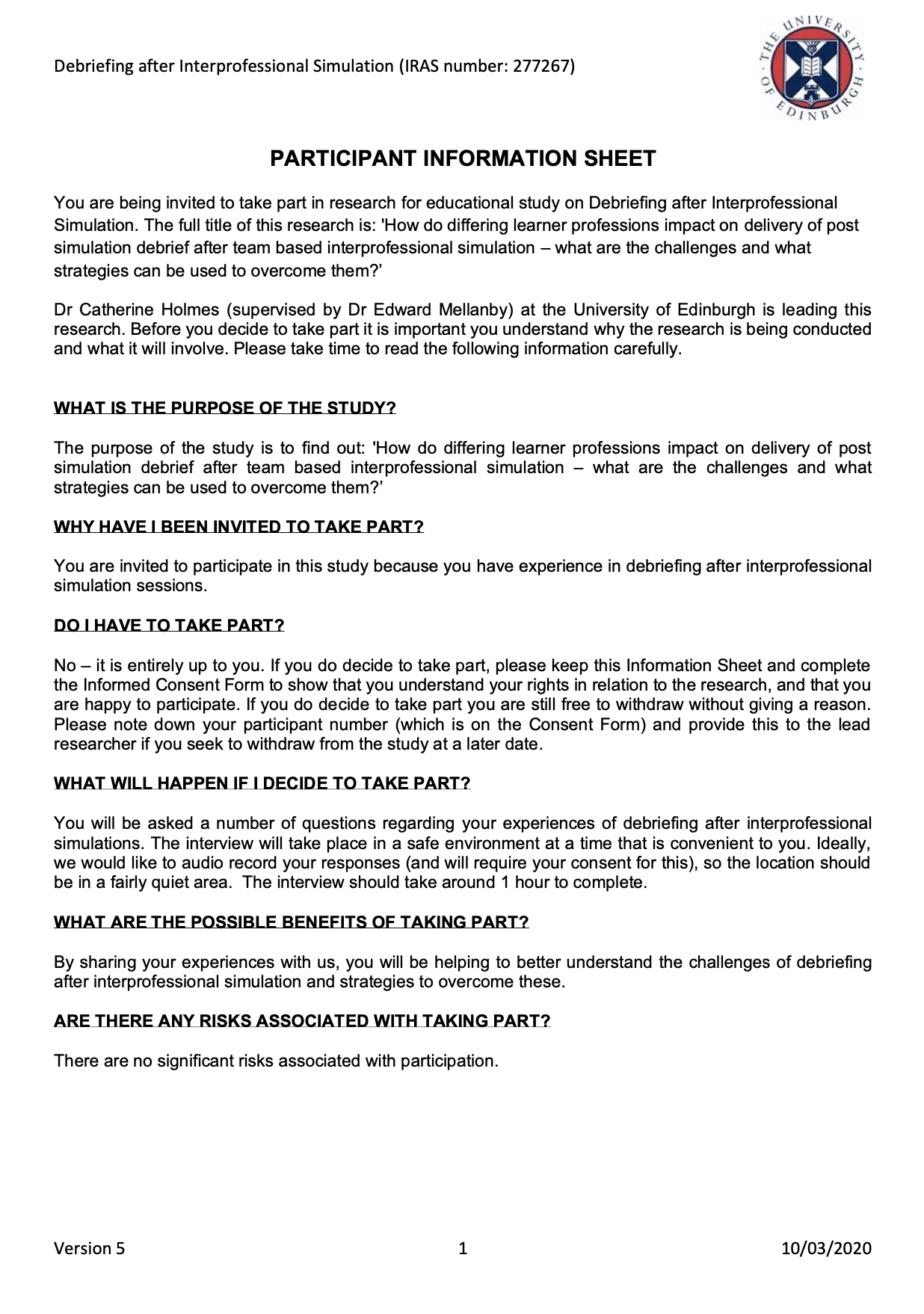
**


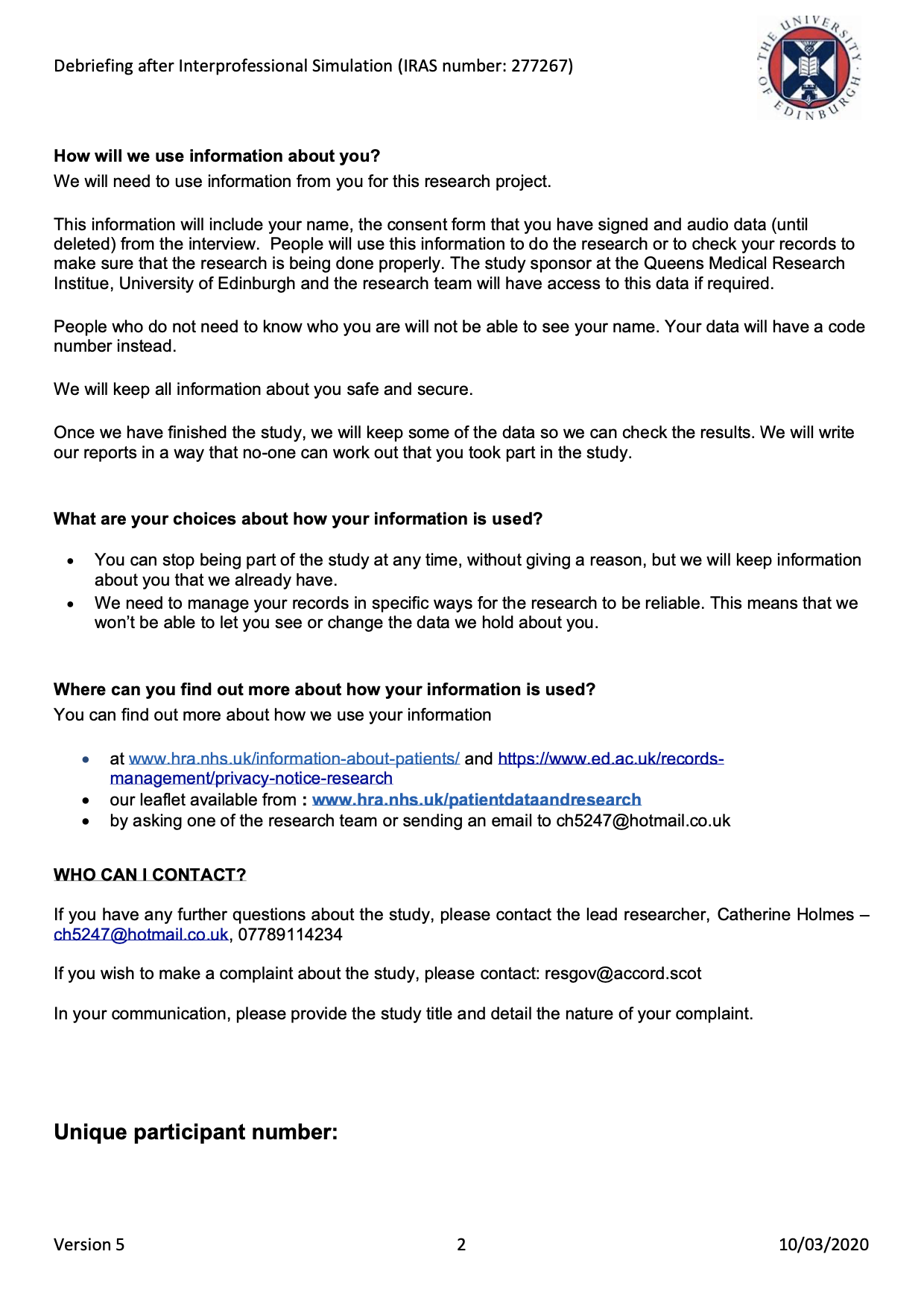

Supplement: Supplementary file 3 — Additional file 3: Appendix 3. Participant information sheet. [file 41077_2022_214_MOESM3_ESM.docx]

**
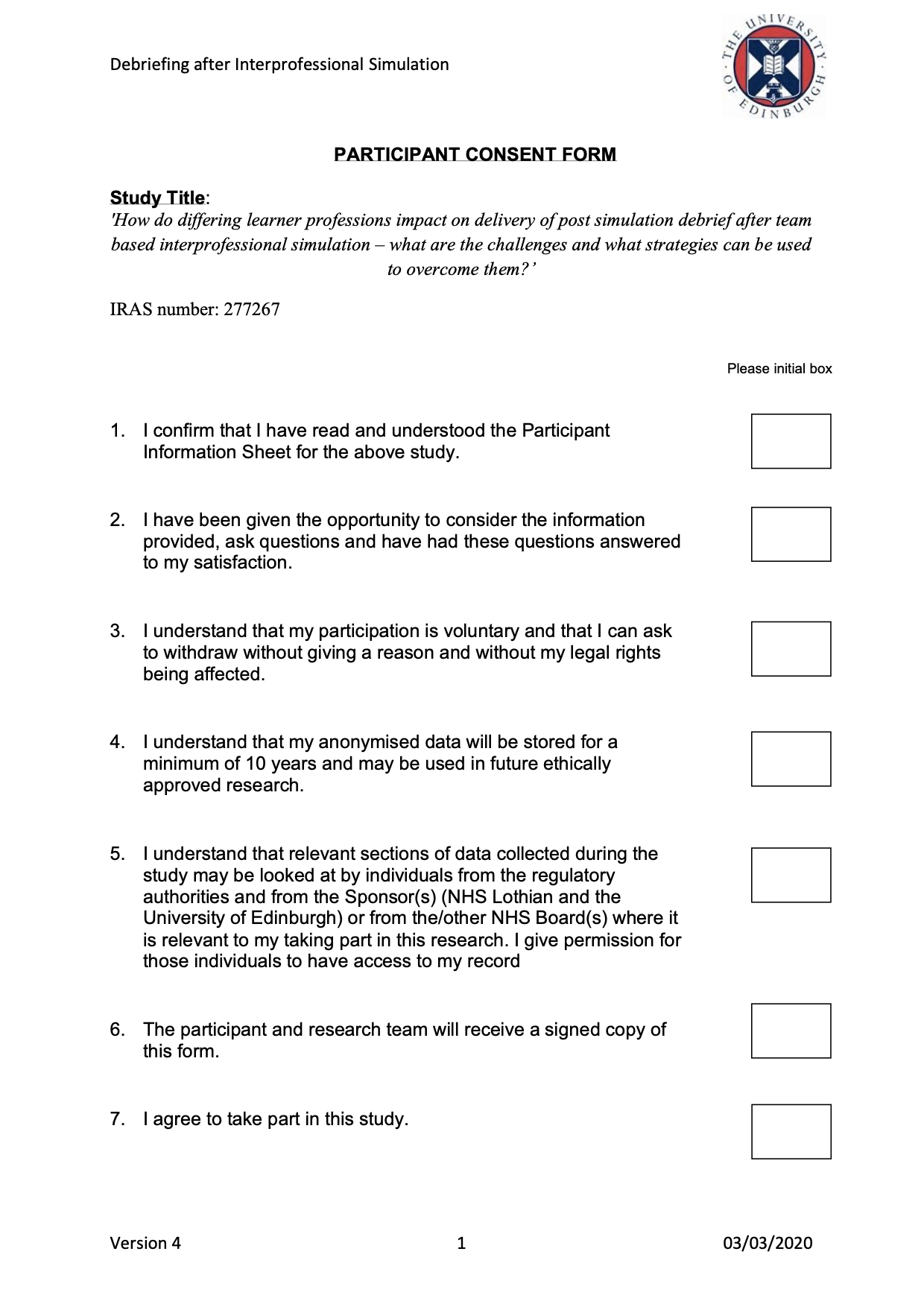
**

**
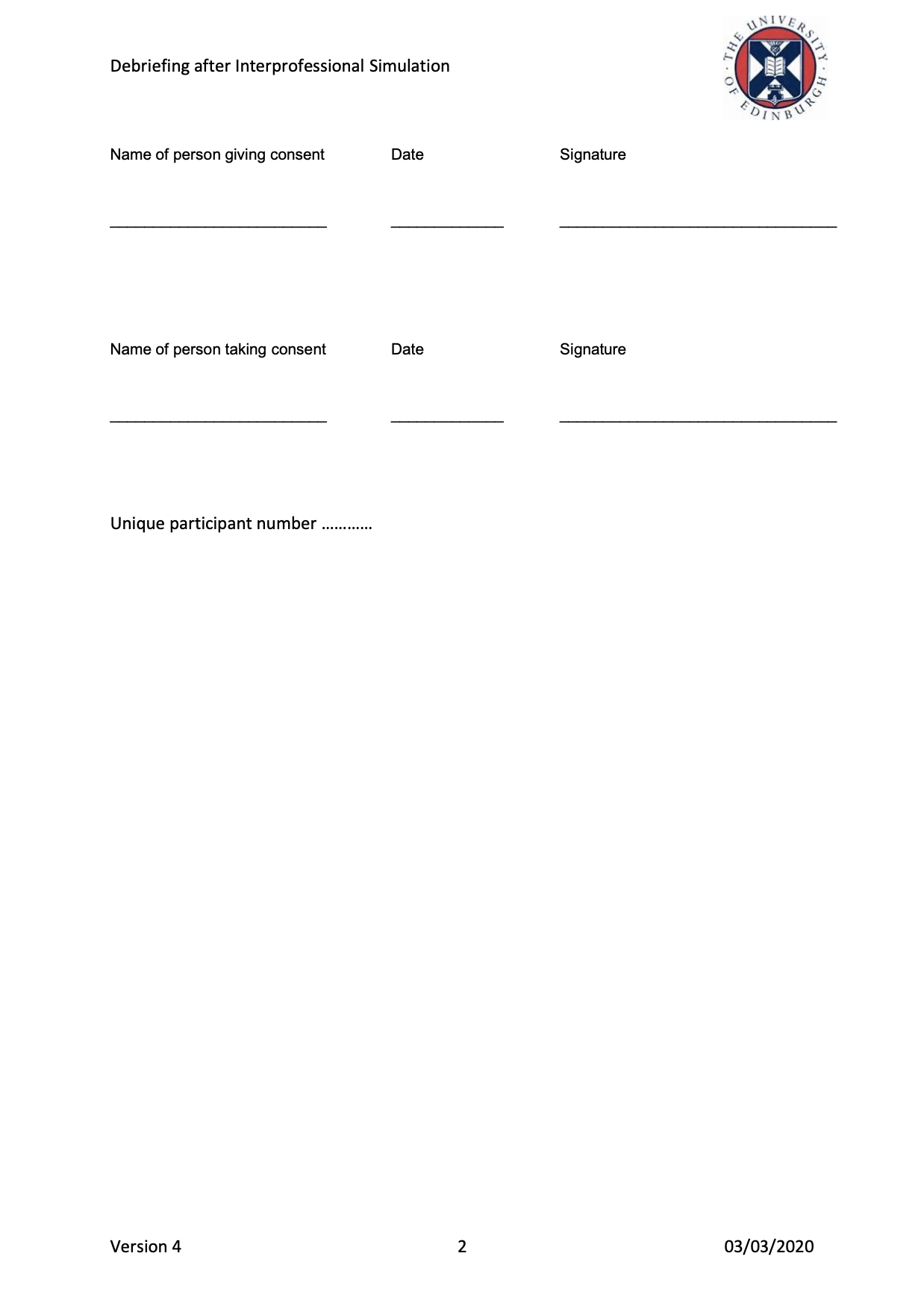
**

Supplement: Supplementary file 4 — Additional file 4: Appendix 4. Participant consent form. [file 41077_2022_214_MOESM4_ESM.docx]

**
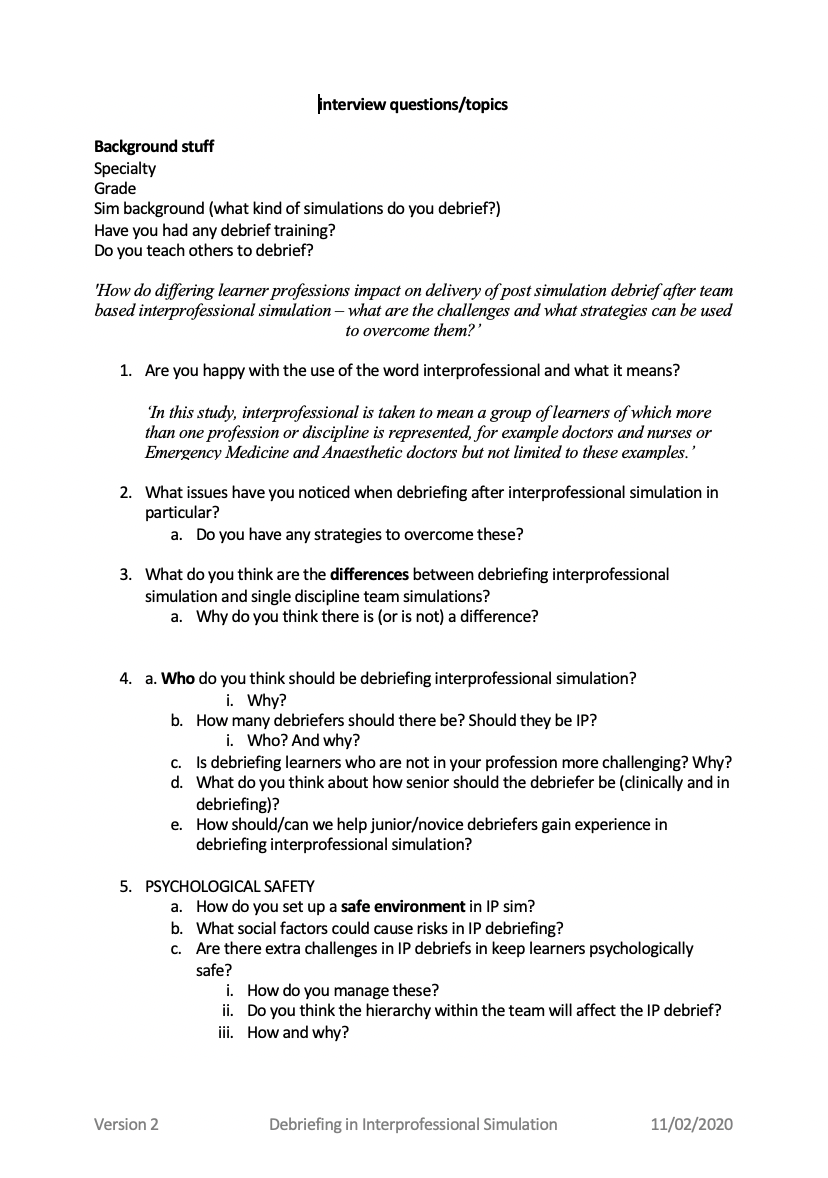
**

**
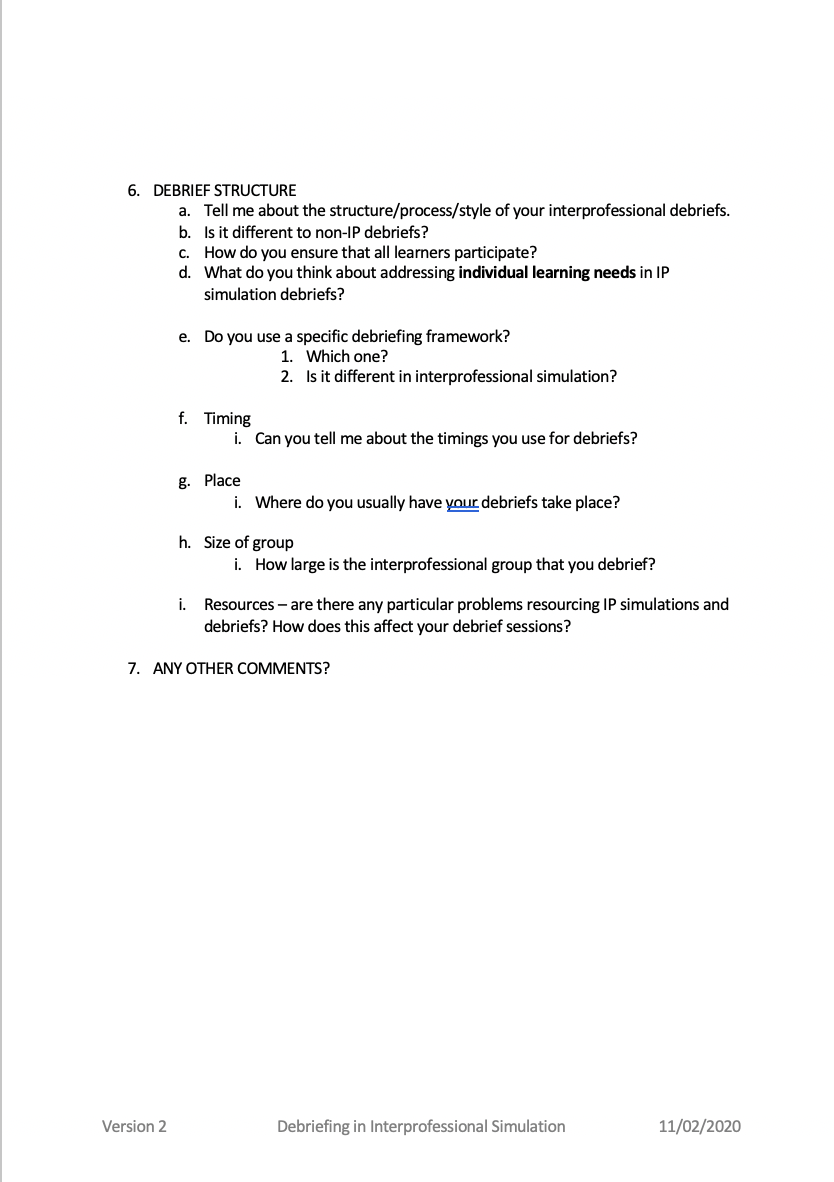
**

Supplement: Supplementary file 5 — Additional file 5: Appendix 5. Interview questions. [file 41077_2022_214_MOESM5_ESM.docx]
